# Impact of COVID-19 on the supply chain of essential health commodities: a mixed method study, in Dar es Salaam, Tanzania

**DOI:** 10.1186/s40545-023-00617-1

**Published:** 2023-09-22

**Authors:** Pius Ipagala, Eulambius M. Mlugu, Rogers Mwakalukwa, Godeliver A. Kagashe

**Affiliations:** 1 Department of Pharmaceutics and Pharmacy Practice, School of Pharmacy, Muhimbili University of Health and Allied Sciences, P.O. Box 65013, Dar es Salaam, Tanzania; 2 Department of Pharmacognosy, School of Pharmacy, Muhimbili University of Health and Allied Sciences, P.O. Box 65013, Dar es Salaam, Tanzania

**Keywords:** COVID-19, Supply chain, Essential health commodities, Tanzania

## Abstract

**Background:**

The outbreak of COVID-19 in the late 2019 led to major global health crises, including morbidities and mortalities. The pandemic has adversely affected the supply chain of essential health commodities globally. However, such data from sub-Saharan Africa including Tanzania are largely limited. We assessed the impact of COVID-19 on the supply chain of essential health commodities in Tanzania.

**Method:**

A cross-sectional study with pragmatic mixed method design was conducted in Dar es Salaam region from January to June 2021. Grounded theory was adopted to purposeful select key informants (*n* = 15) from importers of essential health commodities and local pharmaceutical manufacturers. Community pharmacy dispensers (*n* = 242) were also recruited for the quantitative part of this study. The prices of selected tracer health commodities were extracted from the Tanzania Medicine and Medical Device Authority (TMDA) Regulatory Information Management system. The mean unit prices 1 year before the pandemic were compared with the mean prices 1 year during the pandemic using paired *t* test. Thematic analysis was used for qualitative data.

**Results:**

The information regarding the impact of COVID-19 on the supply chain of essential health commodities was synthesized into three main themes namely, reduced availability of health commodities, increased price of health commodities and increased lead time for imported essential health commodities during COVID-19. Majority (90%) of community pharmacy dispensers reported that COVID-19 reduced the availability of essential health commodities. Azithromycin, Paracetamol, Multivitamin and Vitamin C tablets were the highly demanded products and their mean unit prices increased significantly during COVID-19 as compared to 1 year before the pandemic (p < 0.05).

**Conclusions:**

COVID-19 led to shortage, increased prices and delayed delivery of essential health commodities. This might happen in the future whenever unexpected crises causing disruption in the supply chain occur underscoring the need for the country preparedness measures.

## Background

Essential health commodities are products that meet the top healthcare needs of a population [1]. These products are critical in saving human lives, which sets health supply chains apart from other supply chains [2]. The health supply chain cycle includes selecting health commodities, quantifying the needed amounts, procuring, distributing, and ensuring rational use. In Tanzania, the Medical Stores Department (MSD), a government agency, is responsible for procurement, storage, and distribution of health commodities in the public sector. The MSD receives funding from the government and other donors to procure pharmaceuticals, which are then distributed to health facilities and managed through an electronic Logistic Management Information System (eLMIS) [3]. When health commodities are out of stock at the MSD, health facilities use the private Prime Vendor System (PVS), which is commonly known as JAZIA, to bridge the supply gap [4]. In addition, Tanzania has more than 1,500 private registered retail pharmacies serving the public and about 45 Market Authorization holders importing health commodities to the country [5]. Tanzania like other countries in sub-Saharan Africa imports the majority of its health commodities, including raw materials and finished product [6].

Pandemics and natural disasters pose risks to the health supply chain, causing demand–supply disruptions. The COVID-19 pandemic adversely affected the global health in various ways. To control the effects of COVID-19 pandemic, the World Health Organization (WHO) recommended various strategies including lock down, social distancing, wearing face masks and washing hands with soap and running water or sanitizer. Implementation of the control strategies resulted in ceasing of international flights, closing some manufacturing industries and reducing workers, to comply with social distancing. This caused disruptions in different stages of health commodities supply chain [7] including production, procurement, and distribution, [8, 9]. Albeit, Tanzania was not spared from the effects of the pandemic, there are limited data on the impact of COVID-19 pandemic on the supply chain of health commodities from Tanzania.

The COVID-19 pandemic has resulted in an increased demand for health commodities used to manage COVID-19-related conditions, which may shift the supply chain, affecting the availability and prices of essential health commodities for managing other diseases [10]. This shift may delay country’s efforts to achieve its sustainable development goals.

This study investigated the impact of the COVID-19 pandemic on the supply chain of selected health commodities in Dar es Salaam, Tanzania's largest business city. The findings may inform targeted interventions and preparedness for sustainable supply of essential health commodities.

## Methods

### Study design and setting

This cross-sectional study assessed the impact of COVID-19 on the supply chain of essential health commodities in Dar es Salaam, Tanzania. The study employed pragmatic mixed research methods consisting of qualitative (Key informant interviews) and quantitative (checklist and questionnaire). The study involved key institutions and stakeholders involved in the supply chain of essential health commodities. The key Institutions included Tanzania Medicines and medical Devices Authority (TMDA), local pharmaceutical manufacturers, importers of health care commodities “distributors or wholesale pharmacies” and community retail pharmacies. Dar es Salaam is the largest business city located on the eastern coast of Tanzania. Most pharmaceutical industries, community pharmacies and importers of health commodities are found in the region.

### Study participants recruitment

A total of 242 retail community pharmacies were randomly selected. One staff from each community pharmacy was conveniently recruited for the quantitative part. For qualitative part, a total of 15 participants from local pharmaceutical industries and importers or wholesalers of essential health commodities were purposefully selected based on their positions and experience within the companies. These were CEOs and heads of relevant departments who had worked with companies for more than 2 years.

### Data collection

For qualitative part, we employed semi-structured interview guides to gather information from the key informants. Interview guides were developed based on the knowledge from the literature and experiences of researchers guided by the known supply chain indicators. The questions probed for the general understanding of the COVID-19 pandemic (transmission, prevention, treatment and control strategies). In addition, explored the experiences on the usual operation in the supply chain cycle. One of the questions also explored the participants’ experience and understanding of the impact of COVID-19 on the supply chain of health commodities indicators, such as availability, lead time and price.

A total of 15 in-depth interviews were conducted, among which 12 were conducted among importer or wholesalers of health commodities and three among local pharmaceutical manufacturers. Different interview guides were used for local pharmaceutical manufacturers and importers or wholesalers of health commodities. The interviews were conducted using Swahili language which is native to both participants and researchers. The interviews were conducted in the offices of the KIIs and lasted for about 25–30 min with prior appointments 1 day before the interview. All interviews were audio recorded, and researchers took notes of some important issues including non-verbal to supplement the recorded information. Saturation point was considered to be the 15^th^ interview as there was no further information coming from participants.

For quantitative part, semi-structured investigator centered questionnaire was used to collect data from community retail pharmacy workers. The questions explored the availability, demand patterns and price of essential health commodities in the community. A checklist was also used to collect information regarding the prices (USD) of selected health commodities ordered 1 year before COVID-19 and 1 year during the pandemic. The mean unit price for selected health commodities from January to December 2019 was compared to the mean unit price from March 2020 to February 2021. The source data for this information was the TMDA database known as `Regulatory Information Management system version 2.0 (RIMS 2.0). All the tools were piloted before the study commencement.

### Data analysis

Quantitative data were analyzed by using SPSS software version 25.0. Before analysis, data were cleaned by running frequency distribution to check for data consistency and correctness. Descriptive statistics was used to summarize the data, whereas categorical variables were presented as frequency distributions with percentages, whereas quantitative variables were summarized using measures of central tendency (Mean/median) and measures of variability (Standard deviation/range). The mean prices for selected health commodities before and during the COVID-19 pandemic were compared by using paired *t* test.

A system of quality check was developed to ensure all data received are accurately transcribed and translated. Audio recorded qualitative data were transcribed verbatim. Transcripts were read several times to get sense of the content and the context where they were generated. Thematic analysis was used to analyze the collected information. Initial codes were generated based on the collected data reflecting the research questions. The patterns of codes were evaluated and grouped into the emerged sub-themes and themes which were reviewed and refined. The results were finally summarized based on themes that emerged from the data and illustrated by selected quotes from study participants.

To ensure credibility of the results, we used method triangulation, where interviews and field notes were used for qualitative data collection, whereas questionnaire and register based methods were used for quantitative data. We also employed investigator triangulation where two researchers coded and analyzed the qualitative data. Regular meetings were held during data analysis and through discussion discrepancies were resolved. Sufficient information related to our research question was collected.

### Theoretical framework

The analysis of qualitative data was underpinned by grounded theory, a theoretical framework that involves the construction of hypothesis or construct theory from real world systematically collected and analysed data. Application of this theory provided us with the robust platform to explore the participants’ experience and understanding of the impact of COVID-19 on the supply chain of essential health commodities in Tanzania.

### Ethical considerations

Ethical approval was granted by the Institutional review board of the Muhimbili University of Health and Allied Sciences (MUHAS) (05-2021-605). A permission to conduct the study were requested from the respective institutions. All principles of subject privacy and confidentiality were observed throughout this study. No names of participants were used instead special anonymous study identity codes were used. The audio recorders were kept secured and discarded after the analysis. All participants gave informed consent before commencement of the study.

## Results

### Sociodemographic characteristics of the study participants

A total of 257 participants participated in this study between January to June 2021. Out of 257, 15 participants, three from pharmaceutical industries and 12 from wholesalers or importers were recruited for qualitative part and 242 participants were recruited for the quantitative part. The participants of qualitative part had a mean age with one standard deviation of 35.6 (7.8) years and 13 were males while two were females. About two thirds (69%) of participants in quantitative part were females and 54.1% had a working experience of 2–5 years. Table 1 shows the sociodemographic characteristics of the study participants for quantitative part.

**Table 1:** Demographic information of participants (*n* = 242)

Categories of participants	Frequency	Percentage
Gender		
Male	73	30.2
Female	169	69.8
Working experience		
< 2 years	15	6.2
2–5 years	131	54.1
> 5 years	96	39.7
Education level		
Certificate	129	53.3
Diploma	97	40.1
Degree	14	5.8
Masters or more	2	0.08
Designation/working post		
Dispenser	228	94.2
Manager	12	5
Director	2	0.8

### Impact of COVID-19 on essential health commodities

Three overarching themes emerged from the 15 KIIs conducted among 15 participants. The themes were reduced availability of health commodities during COVID-19, Increased price of health commodities during COVID-19 and Increased Lead time for imported essential health commodities during COVID-19 (Table 2).

**Table 2:** Impact of COVID-19 on essential health commodities

Themes	Sub-themes
Reduced availability of health commodities during COVID-19	Reduced production of health commodities
	Increased demand of some health commodities
	Delayed delivery of health commodities
Increased price of health commodities during COVID-19	Increase in transportation costs
	Increased clearance costs
	Increased price due to Withholding stocks
Increased Lead time for imported essential health commodities during COVID-19	Decreased shipping activities
	Decreased freight and customs activities

### Reduced availability of health commodities during COVID-19

Participants reported activities which led to reduced availability of some essential health commodities during COVID-19 as compared to the times before the pandemic started. The activities were reduced production of health commodities, increased demand of health commodities, and disruptions of transportation.

#### Reduced production of health commodities

Local pharmaceutical manufacturers said that the measures used to control COVID-19 pandemic led to reduced production of health commodities and consequently their availability. To implement the pandemic control measures, manufacturers had to reduce the number of staff in pharmaceutical industries with the aim of maintaining social distance and decongesting pharmaceutical plants as a measure to prevent infection. This has led to slow production and impacted the availability of health commodities in the market.

“……Manufacturing was also taking long because of working shifts to maintain social distance, imagine the plant which was taking 200 to 300 workers now using few people to reduce congestion, must use long period of time to manufacture the required amount of health commodities hence reducing availability in the market” (Participant 6).

Importers of health commodities further mentioned that some countries exporting health commodities were highly affected by the pandemic and the production of such commodities stopped and the importation also ceased due to lockdown and other restrictions imposed to control the pandemic. As a result, the availability of such products in Tanzania were adversely affected.

“…. There were certain times the importation process was ceased and especially from countries which were highly infected by COVID-19, some companies in countries where we import commodities from stopped production due to lockdown” (participant 2).

“…. Availability of some medicines we import decreased as their manufacturers reduced operations especially during the first phase of COVID-19” (Participant 8).

### Increased demand of some health commodities

The importers of health commodities reported that some health commodities were unexpectedly scanty in the market during COVID-19 as compared to before the pandemic. The reduced availability was attributed to increase demand of those products especially those which were used to treat conditions like the symptoms of COVID-19.

“YES, we based on the products used in management of Pneumonia, Upper respiratory infection and Paracetamol for fever; their demand was high, and it was difficulty to get them sometimes” (Participant 6).

Participants added that, before COVID-19 they use to stock sufficient quantities and never run out of stocks, but the demand of these health commodities rose dramatically to more than ten times after the emergency of the pandemic, to the extent of running out of stock.

“…another thing is changes in demand, you find the amount you projected before COVID-19 is 300 units and then someone comes with a demand of 5000 units, so you find running out of stock” (Participant 3).

### Increased demand and reduced Availability of essential health commodities

The quantitative design also revealed that COVID-19 pandemic reduced the availability of health commodities in the community. A total of 216 (89%) out of 242 community retail pharmacy workers reported that COVID-19 reduced availability of health commodities in the market.

The reduced availability was partly attributed to increased demand of some commodities implicated for managing COVID-19-related conditions. Most participants, 92% and 84% mentioned azithromycin and vitamin C, respectively as some of the products whose demand has substantially increased during the COVID-19 as compared to before the pandemic (Table 3).

**Table 3:** Health commodities which were highly demanded during COVID-19 (*n* = 242)

Health commodities	Frequency	Percentage
Azithromycin	222	92
Vitamin C	204	84
Multivitamin	91	38
Prednisolone	74	34
Dexamethasone inj/tab	62	26
Paracetamol	62	26
Zinc	61	25
Aspirin junior	58	24
Amox + clavulanic tabs	54	22
Covidol	38	15
Nimricalf	27	12
Heparin inj	16	7
Cetirizine	15	6
Masks	8	3
Sanitizers	7	3
Calcium Supp	5	2
Celestamine	3	1
Ampicillin + cloxacillin caps	3	1

Participants reported that the highly demanded health commodities were not readily available during the pandemic. More than half of the participants (58%), and 46% reported that vitamin C and multivitamins were not readily available during COVID-19 pandemic. Azithromycin was highly demanded but fairly available (Table 4).

**Table 4:** Health commodities whose availability was reduced by COVID-19 (*n* = 242)

Health commodities	Frequency	Percentage
Vitamin C	141	58
Multivitamins	111	46
Dexamethasone	104	43
Azithromycin	43	18
Aspirin	40	17
Ivermectin	30	12
Colchicine	27	11
Paracetamol	26	11
Zinc	23	10
Replace H	20	8
Examination/surgical gloves	18	7
Prednisolone	18	7
Exam/surgical gloves	10	4
Prednisolone	6	2
Calcium supplements	6	2
Chloroquine tabs	3	1
Amoxicillin + clavulanic acid tablet	3	1

### Increased price of health commodities during COVID-19

Pharmaceutical importers/wholesalers reported that during COVID-19, the interruption of supply chain activities increased importation cost and consequently the price of some essential health commodities. The interrupted activities deduced three subthemes which were the rise in transportation costs, decrease in port clearance services and withholding stocks of health commodities.

#### Increase in transportation costs

Participants mentioned that the transportation costs increased during the pandemic and had serious implication to selling prices of health commodities. They reported that the usual transportation route by sea shipment declined substantially due to lockdown and boarder closures in exporting countries. To ensure availability of some health commodities in the country, they sometimes opted to cargo using aircraft which was more expensive than sea shipping. Eventually, they reviewed their selling price to cater for the added costs.

“On the other side transportation of consignments was very difficult and there are times sea shipment was very slow due to restrictions imposed in exporting countries and you find in such moments when we needed a certain consignment, we opted to cargo only aircraft, this is expensive and has serious implications on our selling prices” (Participant 15).

### Increased clearance costs

It was reported that during the pandemic some clearing and forwarding agents closed their offices and some worked from home to reduce the transmission. This necessitated some importers to use alternative clearing agents who had relatively higher costs to clear their consignments. This added some clearing charges which had impact on the price of health commodities.

“…because there were times when agents’ offices we use to clear and forward our consignments were closed and, so this necessitated us to switch to others who were working, these were charging a lot compared to before and had a lot of works to do, costs of clearance also increased importation costs” (Participant 6).

“It has affected greatly, and there were times clearing agents closed their offices and sometimes were working from home and those who tried to open their office felt to be risking their lives which made them demand high charges all these delayed deliveries of commodities together with reduction of workers at ports of entry to decongest the places as a means to minimize the chances of infection, this imposed difficulties in clearing the consignments” (Participant 8).

### Increased price due to withholding stocks

Participants reported that the pandemic was also viewed as an opportunity to make a profitable business for some suppliers of health commodities used for the COVID-19 symptoms. They added that some suppliers were purposely withholding the stocks of the most demanded products, to create the demand gap and consequently raised their prices.

“Sometimes suppliers of certain products couldn’t release the product or delayed delivering the consignment purposely to make their products less available and specifically those products which were in one way, or another involved in the management of COVID-19 with the intent to raise the price” (Participant 14).

### Increased mean unit prices of selected health commodities

Review of prices of some selected tracer health commodities in the TMDA Regulatory Information Management system revealed that the mean unit prices for some products increased significantly during COVID-19 as compared to 1 year before the pandemic. The significant increase in price was observed for Azithromycin tablets (p = 0.001), Paracetamol tablets (p < 0.001) and multivitamin tablets (p = 0.003). The prices for Examination gloves, Amoxicillin + clavulanic acid tablets, Dexamethasone injection, Heparin injection, Vitamin C and Zinc tablets were also increased as compared to 1 year before the pandemic although statistical significance was not reached (Table 5).

**Table 5:** Mean unit prices of selected essential health commodities before and during COVID-19 outbreak

Tracer health commodities	Mean unit price before COVID-19 (USD)	Mean unit price during COVID-19 (USD)	*P* value
Azithromycin	5.4 ± 0.2	6.129 ± 0.5	0.001
Paracetamol tablets, 500 mg	0.34 ± 0.11	0.58 ± 0.1	< 0.001
Acetylsalicylic Acid tablets, 75 mg	1.22	1.22	
Clopidogrel tablets, 75 mg	1.6	1.6	
Ascorbic acid tablets	1.1 ± 0.15	3.8 ± 0.14	0.57
Multivitamin tablets	2.28 ± 0.06	2.9 ± 0.12	0.003
Heparin injection	2.4 ± 0.08	5.6 ± 0.13	0.237
Prednisolone tablets	0.66	0.66	
Dexamethasone injection	5.3 ± 0.19	9.5 ± 0.12	0.05
Pediatric Zinc tablets	1.5 ± 0.12	1.8 ± 0.15	0.75
Amoxicillin + clavulanic acid tabs	2.85 ± 0.09	4.1 ± 0.08	0.668
Cetirizine 10 mg tablets	0.5 ± 0.16	0.56 ± 0.1	0.077
Ivermectin tablets	1.96	1.96	
Cotton wool (500 g)	23.2	23.2	
Absorbent Gauze	8.57 ± 0.15	8.9 ± 0.17	0.778
Examination gloves	2.18 ± 0.24	6.53 ± 0.13	0.079

### Increased lead time for imported essential health commodities

Lead time refers to the time taken since the order is submitted to the time the consignment is received. The average lead time for delivering of imported health commodities was reported to increase during COVID-19 as compared to the time before the pandemic. The activities which were mentioned as the causes of increased lead time during the pandemic include decrease in shipments and decrease of customs activities.

#### Decreased shipping activities

Importers of health commodities reported that measure taken to contain COVID-19 posed difficulties in shipping and consequently increased the lead time substantially. The control measures in exporting countries lead to boarder closure and airway suspension and resulted in abnormal shipping operations. Participants also mentioned that the decreased production in exporting countries caused insufficient cargos thus delayed shipping and the lead time for delivery of imported health commodities doubled during the pandemic.

“You know, there were boarder closures and airway dumping, especially in countries we import from in such periods and you have an order it was difficult to be attended, then you must have delays and our own experience is that ships were delivering late as up to two times the period before COVID-19” (Participant 9).

“Lead time increased as I said before, during the first wave of COVID-19 cargos reduced to the extent that ships couldn’t make it as it was supposed to be, sometimes ships could travel from one place to another to consolidate cargos, this had impact on delivery time” (Participant 10).

The participants highlighted that before COVID-19 ships took an average of 1 month to arrive and after imposing control restrictions during the pandemic, ships could take up to 2 months.

“There is a difference, there were times the consignment that was to arrive within one month, took up to two months and that’s two times before COVID-19” (Participants 8).

### Decreased freight and customs activities

Participants further said that workforce in ports, clearing and forwarding agents and other key players in movements of consignments was either reduced or working inefficiently as others were working from home, this posed some delays of consignments leading to increased lead time of imported health commodities and pharmaceutical raw materials.

“Yes, some countries where we import medicines closed their boarders, there were no plane entering or leaving those countries, transportation in such countries was difficult, leading to delays, the consignment which could arrive in 20 days, it was taking almost a month and that is even before clearance at our ports and you know during the first wave (2020), we faced a challenge of delayed delivery, suppliers were complaining of delays at the customs as they were not working at their full capacity due to lockdown and other restrictions imposed to fight the pandemic” (Participant 12).

“There are many reasons as I have said before, also during lockdown clearing agents and customs were also working inefficiently causing some delays in getting the ordered consignments” (participant 13).

Local pharmaceutical manufacturers added that during the pandemic the lead time for delivering orders to their customers increased up to three times during the pandemic as compared to before the pandemic due to delayed supply of raw materials from exporting countries.

“There has been a reduced availability of pharmaceutical raw materials, due to reduced production in raw materials producing industries and this is associated with lockdown in exporting countries, this has led to delayed delivery of orders of our customers, you find the order which were formerly attended for one month now are taking up to three months” (Participant 13).

## Discussion

The findings of this study indicate that the COVID-19 pandemic has adversely affected the supply–demand of health commodities in Dar es Salaam Tanzania. This underscores the need for a national wide survey for the country’s specific targeted interventions bearing in mind that the observed impacts may happen in the future whenever unexpected crises causing disruption in the supply chain occur.

According to our findings, the COVID-19 pandemic has had a significant impact on the availability of essential health commodities. The reduced availability of some locally made health commodities was mainly due to the scarcity of raw materials from the source countries. This scarcity could be explained by the COVID-19 shutdown in exporting countries such as China [11] and India [12] which resulted in a shortage of raw materials and finished health products in Tanzania [13]. This is also due to the fact that more 70% of local Pharmaceutical demand including raw materials and finished health commodities are imported [6].

Our results also revealed an increased demand of some health commodities during COVID-19. This increased demand caused the disruption in the supply–demand process and created shortage of essential health commodities. The increased demand for health commodities also caused suppliers to shift their business to the highly demanded health commodities, which sustained the business during the pandemic. This diversion to the needed commodities may have caused shortages of other important health commodities needed for managing poverty-related diseases. Moreover, panic buying of health commodities used for managing COVID-19 increased their demand and contributed to lower availability of such products. Products such as Ascorbic acid, Azithromycin, Multivitamins, Dexamethasone, Acetylsalicylic acid, Ivermectin, Colchicine and Zinc were mentioned to be more demanded and less available during the pandemic. Our findings are comparable to previous studies done in Namibia [14], Saudi Arabia [15], Rwanda [16], United states [17] and European countries [18].

The increased demand for health commodities during the pandemic may have caused an increased flow of substandard health commodities, which need further study. It was also revealed that the increased demand for antibiotics in the community caused dispensing without prescriptions in retail pharmacies, which may pose threats to resistance development. Previous studies reported increased medication errors, treatment cost, adverse drug reactions, and mortalities [19–21] as the effects of drug shortages, which might have also happened during the pandemic. Nevertheless, the impact of COVID-19 reduced availability of essential health commodities on the public health need to be well investigated for future targeted interventions.

Furthermore, our review of prices for some selected health commodities in the TMDA Regulatory Information Management System revealed a significant increase in the prices of some essential health commodities during the COVID-19 pandemic. The significant increase in price was noted for the highly demanded products such as azithromycin, paracetamol and multivitamins. The main reasons for the increased prices were viewed as the increase in transportation cost and clearance cost. Opting for air freight to ensure constant availability of the highly demanded products was the best option due to decreased sea shipment as a result of low number of cargos during the pandemic. Since air freight is relatively expensive than sea shipment, the transportation costs increased and ultimately increased prices for those health commodities. In addition, the reduced port operations caused some consignments to stay at ports longer than usual, leading to added charges and ultimately impacting the price of essential health commodities. The increase in transportation cost during the pandemic was also highlighted in the UNICTAD report on maritime transport [22]. It was also revealed that some clearance agents were not working in fear of the pandemic and those who worked increased the clearance costs resulting in higher prices for the products. Our findings corroborated several other studies [15].

Delayed delivery of imported health commodities during the COVID-19 pandemic was mainly due to the declined shipping, freight, and customs activities. Disrupted production activities in exporting countries caused ships to move from one point to another in search of cargos, causing delays in delivering consignments substantially. Moreover, the customs and clearing services in Tanzania were not functioning at their fully capacity, possibly due to restrictions imposed to fight the pandemic, causing delays in clearance of consignments leading to increased delivery time for imported health commodities. This finding is in agreement with the United Nations Development Program (UNDP) report on socio-economic impact of COVID-19 in Tanzania, which highlighted a declining trend in the number of freight ships in early 2020 and further predicted that Tanzania would face a decline in cargo volumes and subsequent stagnation of domestic logistic services [23].

To our best knowledge, this is the first study to report the impact of COVID-19 on the supply chain of essential health commodities in Tanzania.

The study, however, had some limitations, including the fact that it was conducted in Dar es Salaam region only, thus the results may not be generalized to other regions in the country. The study also did not include some key players in the health supply chain, such as health facilities. In addition, the study focused on essential health commodities, thus data regarding the full impact of COVID-19 on the supply of general health commodities is missing. Further research is needed to investigate the impact of the COVID-19 pandemic on the public health supply chain for future targeted interventions.

## Conclusion

COVID-19 pandemic led to shortage, increased prices and delayed delivery of essential health commodities in Dar es Salaam Tanzania. Leveraging on the lessons, we recommend preparedness measures including attracting investors in pharmaceutical manufacturing focusing on the synthesis of raw materials, stockpiling, and involving pharmacists in forecasting and conducting more research and development in traditional medicines.

## Data Availability

All data generated or analyzed during this study are included in this manuscript.

## Abbreviations

COVID-19: Corona Virus Disease 2019
MSD: Medical Stores Department
WHO: World Health Organization
TMDA: Tanzania Medicine and Medical Device Authority
MUHAS: Muhimbili University of Health and Allied Sciences

## References

[1] Dugani S, Wasan KM, Kissoon N (2018). World Health Organization and essential medicines. J Pharm Sci.

[2] The Lancet H (2019). Essential medicines: a balancing act. Lancet Haematol.

[3] Mwencha M, Rosen JE, Spisak C, Watson N, Kisoka N, Mberesero H (2017). Upgrading supply chain management systems to improve availability of medicines in Tanzania: evaluation of performance and cost effects. Glob Health Sci Pract.

[4] Tibandebage P, Wangwe S, Mackintosh M, Mujinja PGM: Pharmaceutical Manufacturing Decline in Tanzania: How Possible Is a Turnaround to Growth? In: Making Medicines in Africa: The Political Economy of Industrializing for Local Health. Mackintosh M, Banda G, Tibandebage P, Wamae W eds., London: Palgrave Macmillan UK; 2016: 45–64.

[5] Embrey M, Mbwasi R, Shekalaghe E, Liana J, Kimatta S, Ignace G, Dillip A, Hafner T (2021). National Health Insurance Fund’s relationship to retail drug outlets: a Tanzania case study. J Pharm Policy Pract.

[6] Pheage T (2017). Dying from lack of medicines. Africa Renewal.

[7] Wilson MC (2007). The impact of transportation disruptions on supply chain performance. Transp Res E Logist Transp Rev.

[8] Ivanov D (2020). Predicting the impacts of epidemic outbreaks on global supply chains: A simulation-based analysis on the coronavirus outbreak (COVID-19/SARS-CoV-2) case. Transp Res E Logist Transp Rev.

[9] ILO.: The effects of COVID‑19 on trade and global supply chains. https://www.ilo.org/global/research/publications/WCMS_746917/lang--en/index.htm. Accessed 30 Mar 2023.

[10] Emmanuel Awucha N, Chinelo Janefrances O, Chima Meshach A, Chiamaka Henrietta J, Ibilolia Daniel A, Esther Chidiebere N (2020). Impact of the COVID-19 pandemic on consumers' access to essential medicines in Nigeria. Am J Trop Med Hyg.

[11] Chatterjee P (2020). Indian pharma threatened by COVID-19 shutdowns in China. Lancet.

[12] Chris Thomas ND: Reuters. Global supplier India curbs drug exports as coronavirus fears grow. 2020.

[13] Guerin PJ, Singh-Phulgenda S, Strub-Wourgaft N (2020). The consequence of COVID-19 on the global supply of medical products: Why Indian generics matter for the world?. F1000Res.

[14] Tirivangani T, Alpo B, Kibuule D, Gaeseb J, Adenuga BA (2021). Impact of COVID-19 pandemic on pharmaceutical systems and supply chain - a phenomenological study. Explor Res Clin Soc Pharm.

[15] Aljadeed R, AlRuthia Y, Balkhi B, Sales I, Alwhaibi M, Almohammed O, Alotaibi AJ, Alrumaih AM, Asiri Y (2021). The impact of COVID-19 on essential medicines and personal protective equipment availability and prices in Saudi Arabia. Healthcare.

[16] Uwizeyimana T, Hashim HT, Kabakambira JD, Mujyarugamba JC, Dushime J, Ntacyabukura B, Ndayizeye R, Adebisi YA, Lucero-Prisno DE (2021). Drug supply situation in Rwanda during COVID-19: issues, efforts and challenges. J Pharm Policy Pract.

[17] FDA.: Drug shortages. https://www.fda.gov/drugs/drug-safety-and-availability/drug-shortages. Accessed 30 Mar 2023.

[18] EMA.: Reflection paper on forecasting demand for medicinal products in the EU/EEA. https://www.ema.europa.eu/en/documents/other/reflection-paper-forecasting-demand-medicinal-products-eu/eea_en.pdf. Accessed 30 Mar 2023.

[19] Badreldin HA, Atallah B (2021). Global drug shortages due to COVID-19: Impact on patient care and mitigation strategies. Res Social Adm Pharm.

[20] Walker J, Chaar BB, Vera N, Pillai AS, Lim JS, Bero L, Moles RJ (2017). Medicine shortages in Fiji: a qualitative exploration of stakeholders' views. PLoS ONE.

[21] McLaughlin M, Kotis D, Thomson K, Harrison M, Fennessy G, Postelnick M, Scheetz MH (2013). Effects on patient care caused by drug shortages: a survey. J Manag Care Spec Pharm.

[22] UN.: COVID-19 and maritime transport: Impact and responses. 2020. https://unctad.org/publication/covid-19-and-maritime-transport-impact-and-responses. Accessed 30 Mar 2023.

[23] UN.: Socio-Economic Impact of COVID-19. https://www.undp.org/coronavirus/socio-economic-impact-covid-19. Accessed 29 Mar 2023. 2020.

